# Association between SNAP25 and human glioblastoma multiform: a comprehensive bioinformatic analysis

**DOI:** 10.1042/BSR20200516

**Published:** 2020-06-09

**Authors:** Cheng Yu, Jianxing Yin, Xiefeng Wang, Lijiu Chen, Yutian Wei, Chenfei Lu, Yongping You

**Affiliations:** Department of Neurosurgery, The First Affiliated Hospital of Nanjing Medical University, Nanjing 210029, Jiangsu, China

**Keywords:** Glioblastoma multiforme, PPI, Prognosis, SNAP25, TCGA, WGCNA

## Abstract

**Background**: Glioblastoma multiforme (GBM) is a most common aggressive malignant brain tumor. In recent years, targeted therapy has been increasingly applied in GBM treatment.

**Methods**: In the present study, GSE22866 was downloaded from gene expression omnibus (GEO). The genomic and clinical data were obtained from TCGA. The differentially expressed genes (DEGs) were identified and functional analysis was performed using clusterprofiler. Then, the co-expression network for the DEGs was established using the “WGCNA” package. Next, the protein–protein interaction (PPI) was assessed using Search Tool for the Retrieval of Interacting Genes Database (STRING) and hub modules in Cytoscape were screened. The Venn diagram was plotted to showcase the overlapped hub DEGs in PPI network and TCGA. Univariate and multivariate Cox proportional hazards regression analyses were performed to predict the risk score of each patient. Validations of the hub gene were completed in other databases.

**Results**: Functional analysis of the DEGs verified the involvement of DEGs in growth factor binding and gated channel activity. Among the 10 GBM-related modules, the red one displayed the strongest tie with GBM. VAMP2 was filtered out as the most intimate protein. The PPI network and TCGA were comprehensively analyzed. Finally, SNAP25 was identified as a real hub gene positively correlated with GBM prognosis. The result was validated by GEPIA, ONCOMINE database and qRT-PCR.

**Conclusions**: SNAP25 might act as a GBM suppressor and a biomarker in GBM treatment.

## Introduction

Glioblastoma multiforme (GBM) is a most common aggressive malignant brain tumor characterized by poor clinical outcome and short survival time. The median survival time of GBM is 14–15 months, with a 10% probability of 5-year-survival [1]. So far, the most effective therapy for glioblastoma multiforme is surgery combined with adjuvant chemoradiotherapy [2].

Currently, bioinformatics analysis is being widely replicated in the field of cancer research, which saves the necessity of conducting experiments [3,4]. Both GEO and TCGA databases can promote the study of tumors. For example, using TCGA database, Omer et al. reported that cholesterol homeostasis took a critical position in the development of cancers [5]. Wang et al. revealed that ASPM might arouse cirrhosis and subsequent hepatocellular carcinoma [6]. In this research, GEO and TCGA were used to define differentially expressed genes (DEGs). Next, PPI and co-expression networks were constructed to identify the hub genes. Finally, cox prognosis analysis and website validation were conducted to identify the hub gene which might affect GBM.

## Materials and methods

### Data collection and preprocessing

**Figure 1:**
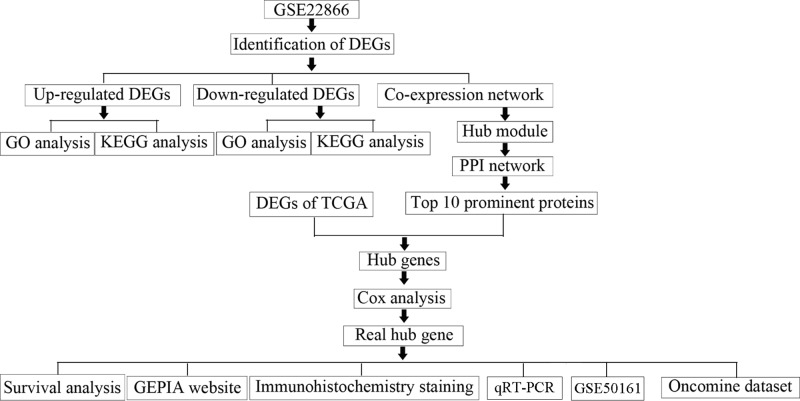
Flow diagram of study

Original data were collected from GSE22866 and TCGA. The expression profiles of related genes were downloaded from the Gene Expression Omnibus (GEO) database (http://www.ncbi.nlm.nih.gov/geo/). GSE22866 data [7] including the information of 40 tumor tissue samples and 6 normal tissue samples, were processed by Agilent-014850 Whole Human Genome Microarray 4×44K G4112F. Robust multi-array average (RMA) approach was adopted for background correction and normalization [8]. The original GEO data were then transformed into expression values using affy R package [9]. The genomic and clinical data of GBM were downloaded from TCGA (https://cancergenome.nih.gov/) and the RNA sequencing data from Illumina Hi-SeqRNA-Seq platform. The flow chart of the whole research is shown in Figure 1.

### Differentially expressed genes (DEGs)

The “limma” R language package [10] was utilized to search the DEGs in GEO data. The adjusted *P*<0.05 and |log2fold change (FC)| ≥1 were chosen as the cut-off criteria. For TCGA data, edge R package was used for DEGs screening [11]. FDR<0.05 and |log2fold change (FC)| ≥ 2 were set as cut-offs. Adjusted *P*-value (adj. p) and FDR were applied to address false-positives. Online Wayne diagram was used to identify the DEGs simultaneously found in CGA and PPI networks. Heatmap was generated using R 3.4.4 [12].

### GO term and KEGG pathway enrichment analysis

The cluster profiler package [13] was used for GO and KEGG analyses. A *P*-value <0.05 indicated the presence of significant enrichment.

### PPI network analysis

Search Tool for the Retrieval of Interacting Genes Database (STRING) (http://www.string-db.org/) was used for PPI between DEGs [14]. The results were visually converted using Cytoscape software. Plug-in Molecular Complex Detection(MCODE) was used to search clusters [15]. Parameters: degree cut-off = 2, node score cut-off = 0.2, *k*-core = 2, and max. depth = 100. Next, functional analysis of the hub genes was performed using the cluster profiler.

### Co-expression network creation

First, the expression profiles of the DEGs were evaluated to verify whether they were associated. Next, the “WGCNA” package in R software was run to construct a co-expression network of DEGs [16,17]. The Pearson’s correlation matrices were both functioned for all pair-wise genes. After that, a power function amn = |cmn|β (cmn = Pearson’s correlation between gene m and gene n; amn = adjacency between gene m and gene n) was utilized to erect a weighted adjacency matrix. A soft-thresholding parameter β was used to emphasize strong correlations and penalize weak correlations. Then, the adjacency of each gene was converted to topological overlap matrix (TOM) to its connectivity or similarity with others. With a minimum size (gene group) of 100 genes in each cluster, dendrograms were created to classify the genes into different modules. Then, the dissimilarity of module eigengenes was calculated to identify the relevant module that had an impact on GBM.

### Construction of a prognostic signature

The hub genes were subjected to univariate Cox proportional hazards regression analysis. Prognosis-related DEGs were defined using *P*<0.05 as the cut-off. Next, a multivariate Cox proportional hazards regression model was constructed based on the screened hub genes. Cox proportional hazards regression with a *P*<0.05 was constructed to predict the risk score of each patient carrying the DEGs. Patients were divided into a low-risk group and a high-risk group according to the mean risk score. Kaplan–Meier curve analysis was conducted to compare the survival time of the two groups. *P*<0.05 was considered statistically significant. Receiver operating characteristic (ROC) curve analysis was also performed to estimate the value of the signature in predicting 5-year survival. The prognostic value was presented as area under the ROC curve, along with sensitivity and specificity.

### Validation of hub genes

The hub genes were validated in GEPIA (Gene Expression Profiling Interactive Analysis) [18]. The Human Protein Atlas (HPA) (http://www.proteinatlas.org/), TCGA dataset, GSE50161 dataset (34 glioblastoma multiforme samples and 13 normal samples) and ONCOMINE databset were used to validate the expression of the real hub gene [19–21].

### Preparation for human GBM samples

Informed consents were obtained from all the patients. The study was approved by the Institutional Review Board of Nanjing Medical University. GBM tissue was collected and immediately stored in an environment at −80°C. From June 2017 to January 2019, 30 GBM tissues and 30 normal brain tissues were prepared in the Department of Neurosurgery of the First Affiliated Hospital of Nanjing Medical University.

### Quantitative real-time RT-PCR (qRT-PCR) analysis

Total RNA was extracted using TRizol reagent (Thermo Fisher Scientific, Waltham, MA, U.S.A.). The integrity of the isolated RNA was assessed using Agilent Bioanalyzer 2100 with RNA 6000 Nano kit (Agilent Technologies, Santa Clara, CA, U.S.A.). The high-capacity DNA reverse transcription kits (Thermo Fisher Scientific) was used for the transformation of single-stranded complementary DNA from RNA. Real-time quantification was performed using the SYBR Green PCR kit (Thermo Fisher Scientific). The cycle threshold (Ct) of each gene was recorded. The relative expression of SNAP25 was calculated using the 2∧−ΔΔCt method (ΔCt  =  Ct_target gene_ − Ct_internal control_). Forward Primer of BCHE was “TCGTGTAGTGGACGAACGG”. Reverse Primer of BCHE was “TCTCATTGCCCATATCCAGGG”.

### Statistical analyses

All analyses were conducted for three times. Representative data were collected. Two-tailed Student’s *t*-test was utilized to detect the differences between the subgroups. Statistical analysis was performed via SPSS 16.0, graphpad and R software 3.4.4. Statistical significance was set at probability values of *P*<0.05.

## Results

### DEGs and their functions

**Figure 2:**
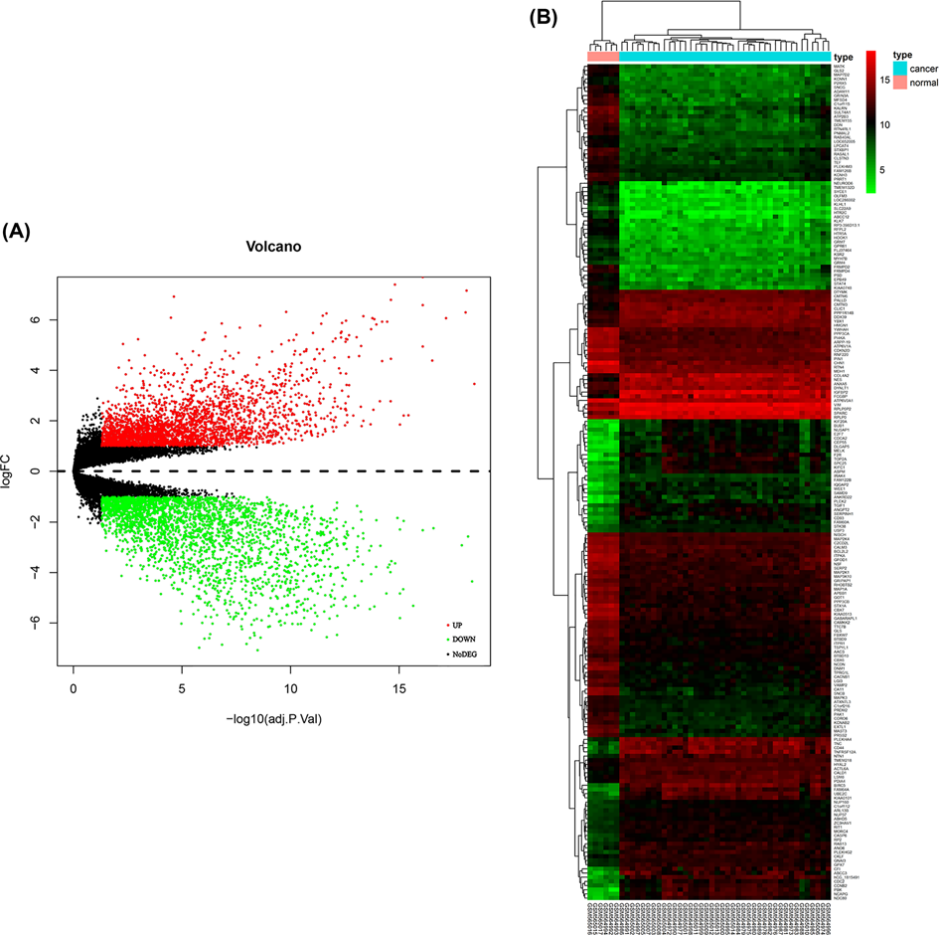
Identification of DEGs in GBM and the enrichment of these genes **Note:** (**A**) Volcano plot of all DEGs. (**B**) Heatmap of all DEGs.

**Figure 3:**
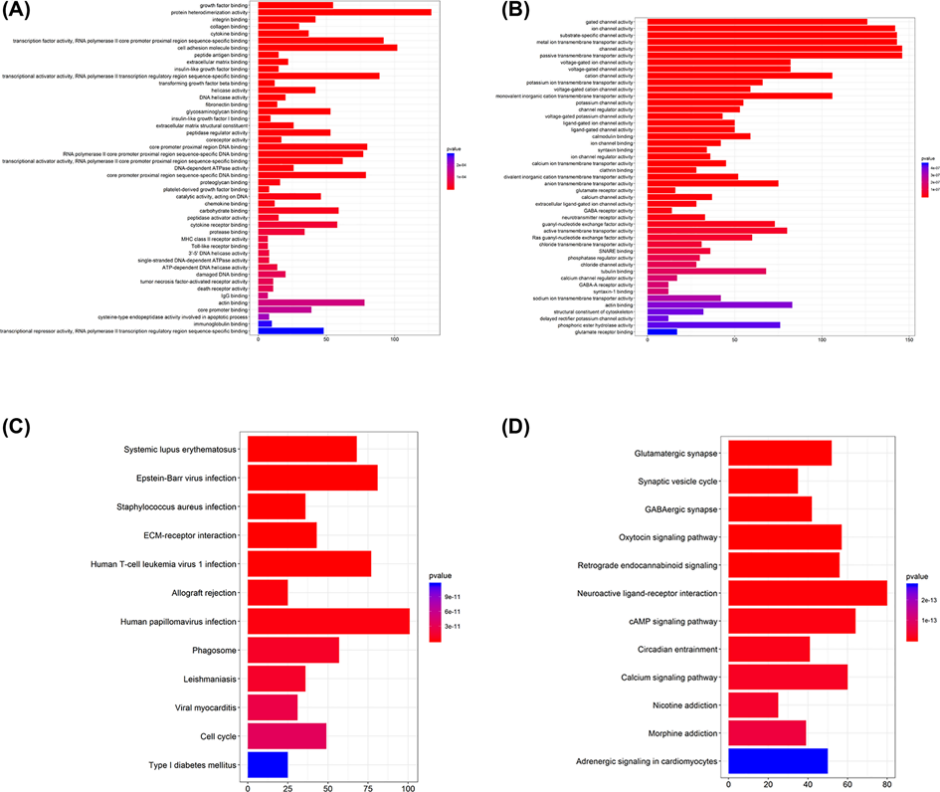
Functional analysis of the DEGs **Note:** (**A**) Up-regulated DEGs with fold change>1 in GO analysis. (**B**) Down-regulated DEGs with fold change > 1 in GO analysis. (**C**) Up-regulated DEGs with fold change > 1in KEGG analysis. (**D**) Down-regulated DEGs with fold change > 1 in KEGG analysis.

A total of 5505 DEGs, including 2817 up-regulated genes and 2688 down-regulated genes in glioblastoma multiforme samples, were screened out (Figure 2A,B). In GO analysis, the up-regulated genes were mostly involved in growth factor binding, protein heterodimerization activity, integrin binding, collagen binding, cytokine binding, transcription factor activity, RNA polymerase II core promoter proximal region sequence-specific binding, cell adhesion molecule binding, peptide antigen binding, extracellular matrix binding, insulin-like growth factor binding, transcriptional activator activity, RNA polymerase II transcription regulatory region sequence-specific binding, transforming growth factor beta binding, helicase activity and DNA helicase activity (Figure 3A); the down-regulated genes were highly involved in gated channel activity, ion channel activity, substrate-specific channel activity, metal ion transmembrane transporter activity, channel activity, passive transmembrane transporter activity, voltage-gated ion channel activity, voltage-gated channel activity and cation channel activity (Figure 3B). According to KEGG results from profiler package [22], the up-regulated genes were mostly enriched in systemic lupus erythematosus, Epstein–Barr virus infection and staphylococcus aureus infection (Figure 3C); the down-regulated genes were mostly involved in glutamatergic synapse, synaptic vesicle cycle and gabaergic synapse (Figure 3D).

### Weighted co-expression network construction and analysis

**Figure 4:**
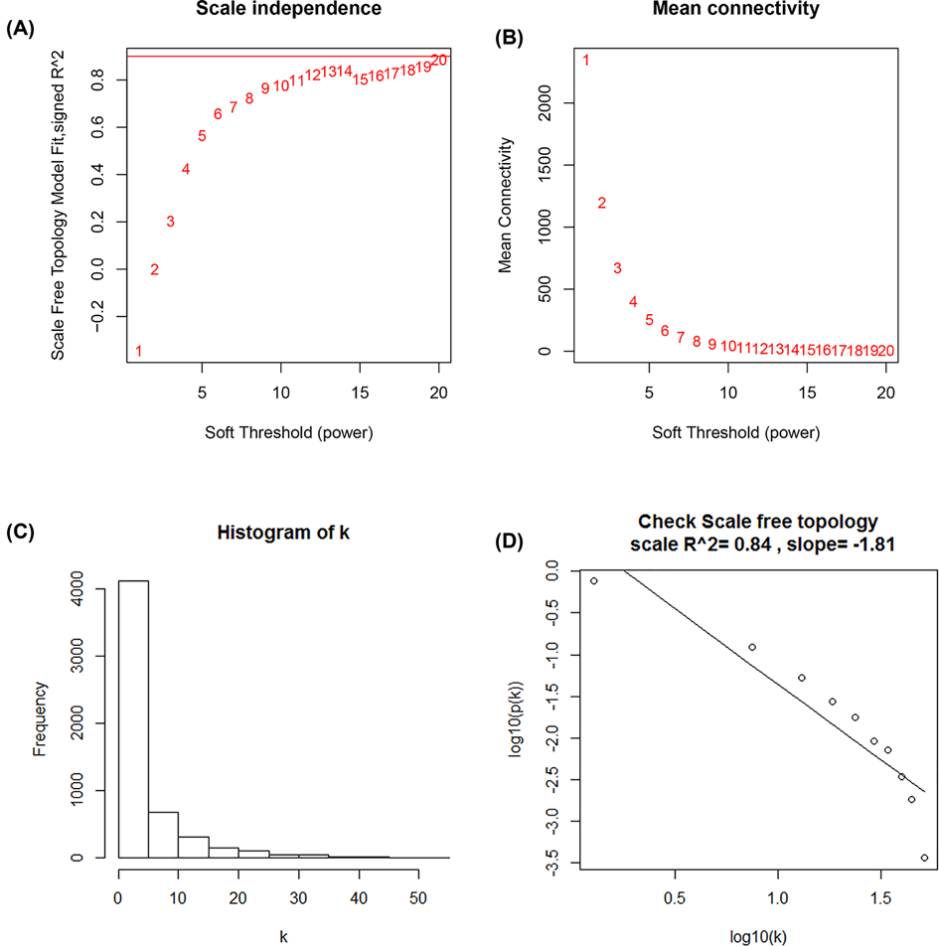
Determination of soft-thresholding power in WGCNA **Note:** (**A**) Analysis of the scale-free fit index for various soft-thresholding powers (β). (**B**) Analysis of the mean connectivity for various soft-thresholding powers. (**C**) Histogram of connectivity distribution when β = 19. (**D**) Checking the scale-free topology when β = 19.

**Figure 5:**
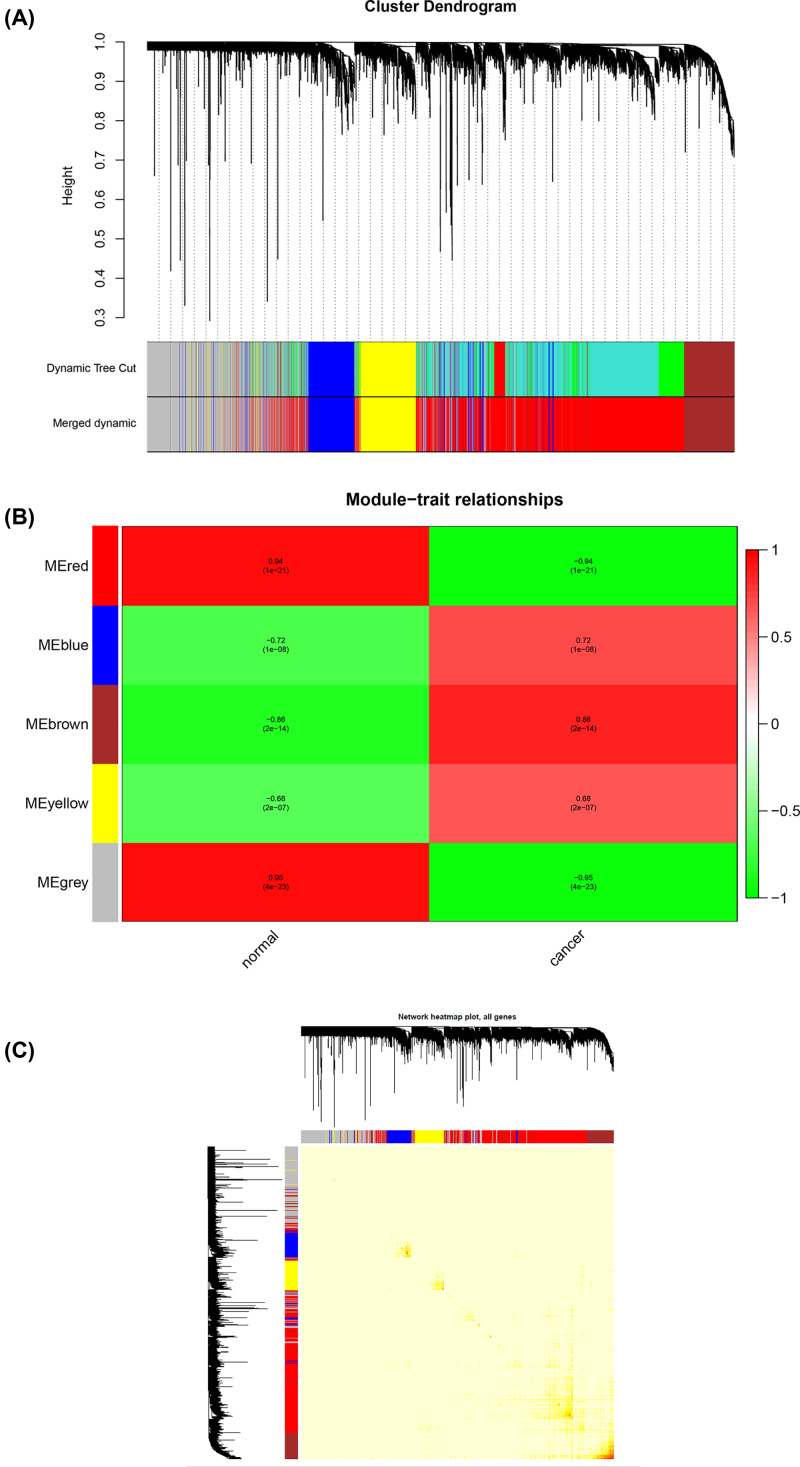
Hub module selection **Note:** (**A**) Dendrogram of all DEGs clustered based on a dissimilarity measure (1-TOM). (**B**) Correlation between modules and traits. The upper number in each cell refers to the correlation coefficient of each module in the trait, and the lower number is the corresponding *P*-value. The redmodule is the most relevant modulewith cancer traits. (**C**) A heatmap of all genes. The intensity of the red color indicates the strength of the correlation between pairs of modules on a linear scale.

**Figure 6:**
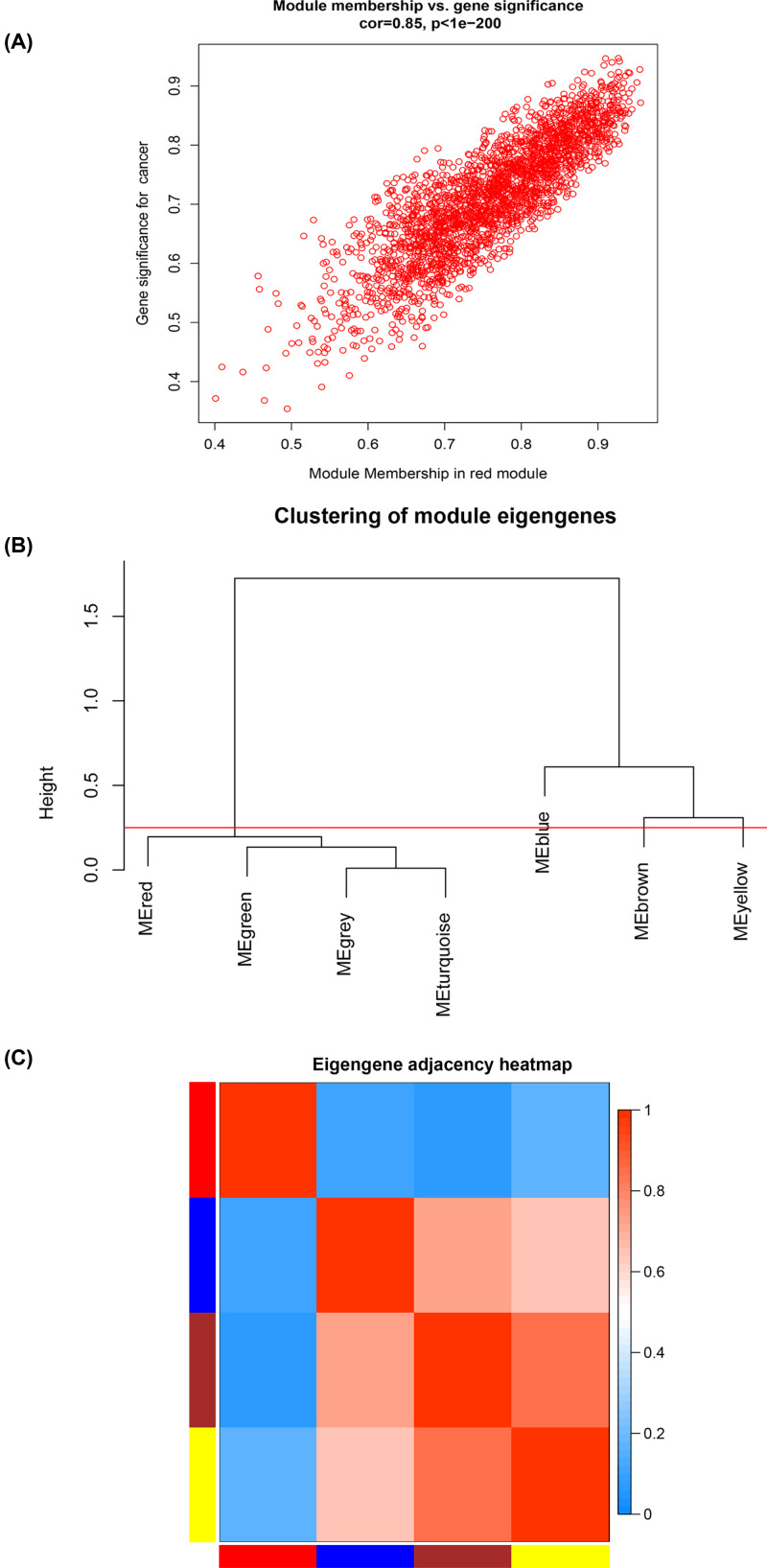
Selection of hub genes in hub modules **Note:** (**A**) A scatter plot of GS for GBM versus the MM in the red module. The intramodular analysis shows the red module contains genes with high correlation with GBM, with *P*<1e-200 and correlation = 0.85. (**B**) Dendrogram of consensus module eigen genes obtained by WGCNA on the consensus correlation. The red line is the merging threshold, and groups of eigen genes below the threshold represent modules in which expressions profiles should be merged due to their similarity. (**C**) Dendrogram of merged module eigen genes obtained by WGCNA. Heatmap plot of the adjacencies of modules. Red represents high adjacency (positive correlation) and blue represents slow adjacency (negative correlation).

The expression data matrix of GSE22866 and the scale-free network were constructed (Figure 4A–D). Seven modules containing highly related genes were excavated (Figure 5A). When MEDissThres was set as 0.25, the seven modules were merged into five (Figure 6B). Module blue was composed of 615 genes, module brown of 604 genes, module grey of 1277 genes, module red of 2434 genes, and module yellow of 575 genes. An intramodular analysis of GS and MM of the genes in the 7 modules was followed. The results showed that among the top 5 modules, the 2434 genes in the red module tend to be remarkably correlated with tumor (Figure 5B) and all genes were identified for the heatmap (Figure 5C). Defined by module connectivity, measured by absolute value of the Pearson’s correlation (cor.geneModuleMembership> 0.8) and cancer trait relationship, measured by absolute value of the Pearson’s correlation (cor.geneTraitSignificance> 0.2), 600 hub genes in the red module were selected for further research (Figure 6A). Among all the modules, module red has the highest negative correlation with the cancer (Figure 5C).

Interestingly, the five modules showed overlap (Figure 6B,C). In general, the five clusters could be classified into two, each containing three branches.

### PPI network and cluster analysis

**Figure 7:**
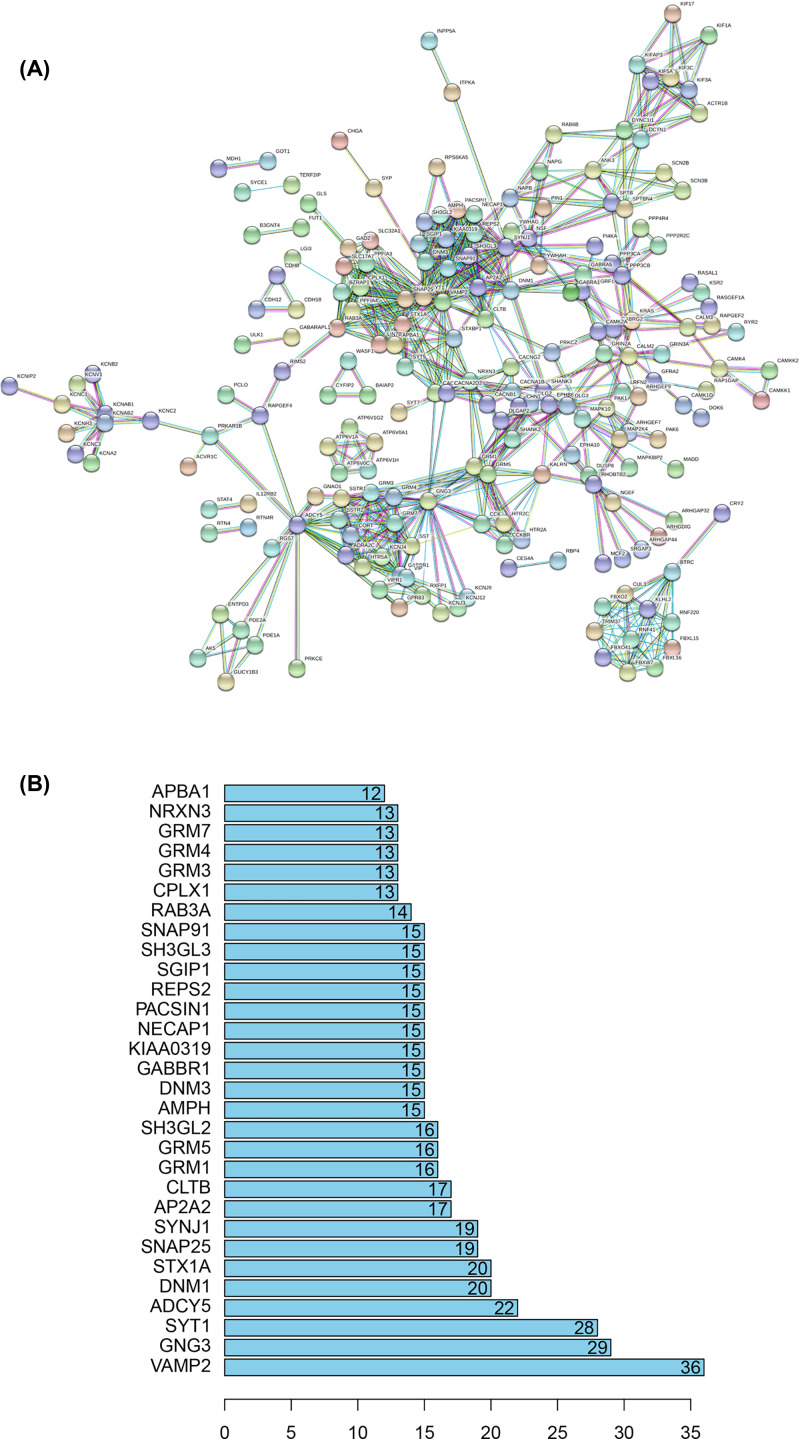
Cluster analysis of the PPI network **Note:** (**A**) A total of 600 hub genes belonging to the red module are filtered into the PPI network complex that contains 586 nodes and 668 edges. (**B**) Histogram of key proteins. The *y*-axis representsthe name of genes, the *x*-axis representsthe number of adjacent genes, and the height isthe number of gene connections.

**Figure 8:**
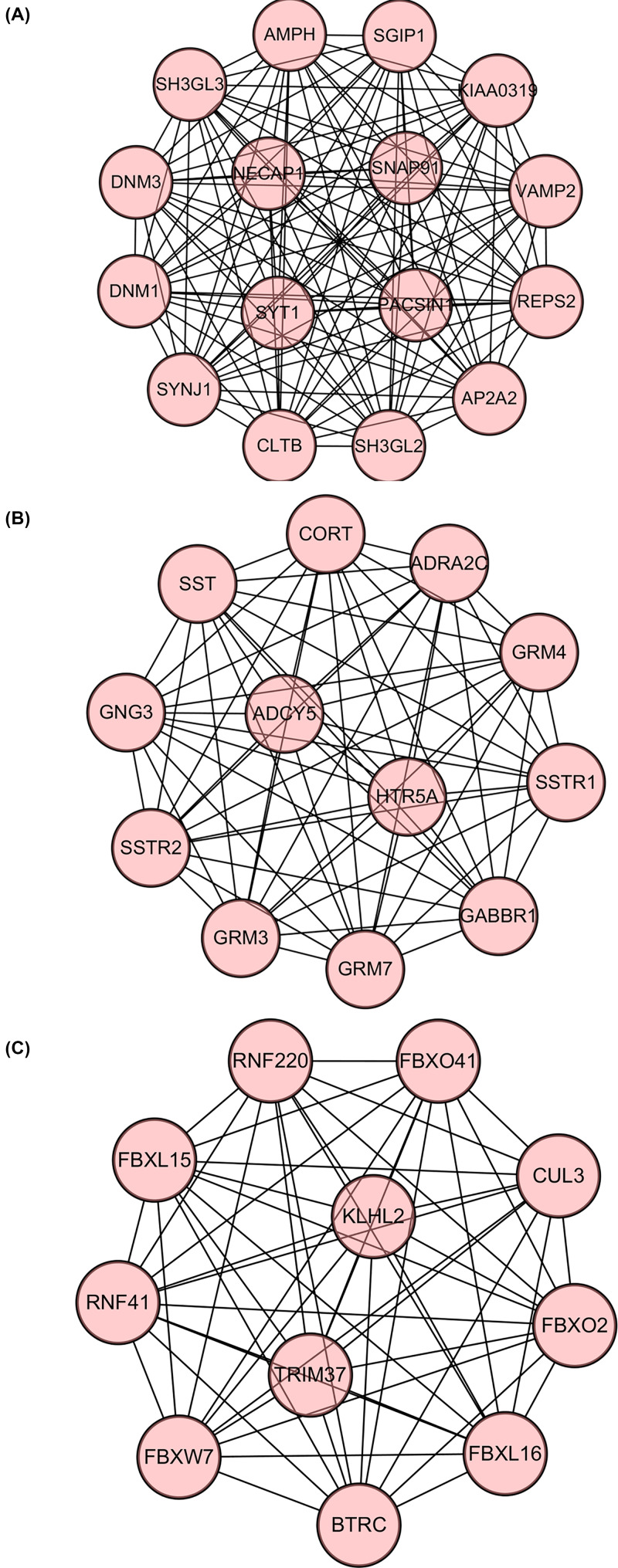
Module analysis of PPI network **Note:** (**A**) Module rank 1. This cluster contains 16 nodes and 120 edges and has the highest score among the clusters. (**B**) Module rank 2. This cluster contains 12 nodes and 66 edges and has the second highest score among the clusters. (**C**) Module rank 3. This cluster contains 11 nodes and 55 edges and has the third highest score among the clusters.

Via the STRING website, 600 hub genes of the red module (including 586 nodes and 668 edges) were submitted into the DEGs PPI network (Figure 7A). The scores of 15 clusters were calculated (k-core = 2). Among them, cluster 1, which contained 16 nodes and 120 edges, was given the highest score (Figure 8A), suggesting that these 16 DEGs might play a critical role in glioblastoma multiforme. Cluster 2, which contained 12 nodes and 66 edges, was given the second highest score (Figure 8B). Cluster 3, which contained 11 nodes and 55 edges, was given the third highest score (Figure 8C). The genes in cluster 2 and cluster 3 might also be associated with glioblastoma multiforme.

**Figure 9:**
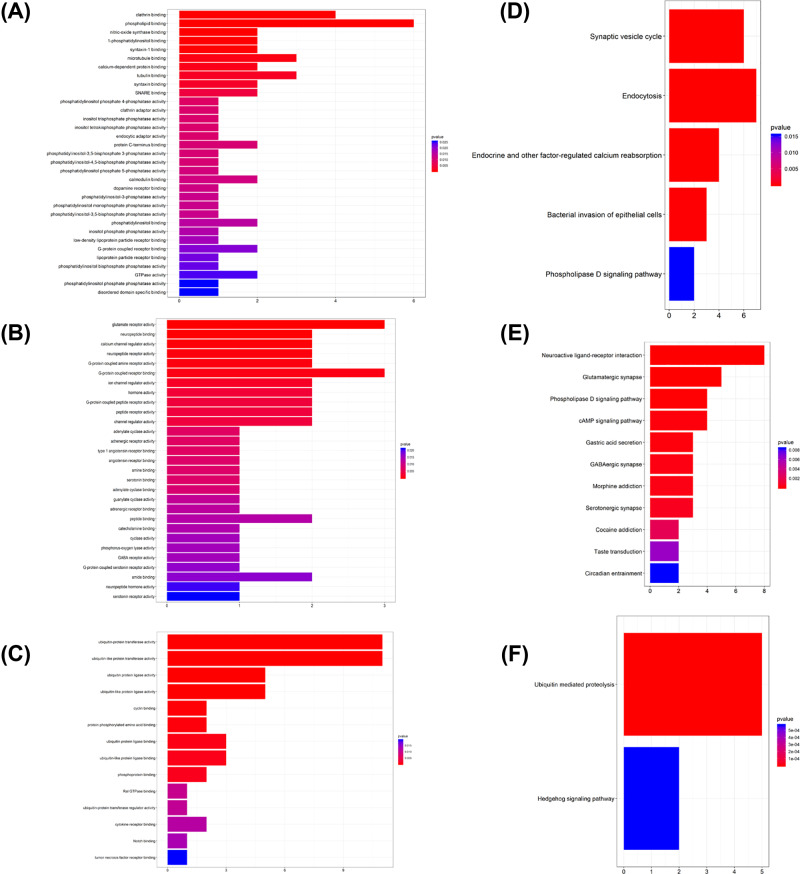
GO and KEGG analyses of the hub modules **Note:** (**A**) GO analysis of module 1. (**B**) GO analysis of module 2. (**C**) GO analysis of module 3. (**D**) KEGG analysis of module 1. (**E**) KEGG analysis of module 2. (**F**) KEGG analysis of module 3.

Plug-in MCODE was used for the top three significant modules of the PPI network. Functional analysis was performed for the three clusters. GO and KEGG analyses were performed by clusterprofiler (Figure 9A–F).

Via PPI analysis, 30 prominent proteins were identified. Among them, VAMP2, which contacted 36 nodes, was considered as the most important protein (Figure 7B). The top 10 prominent proteins were selected for the next step of the study.

### Hub genes validation

**Figure 10:**
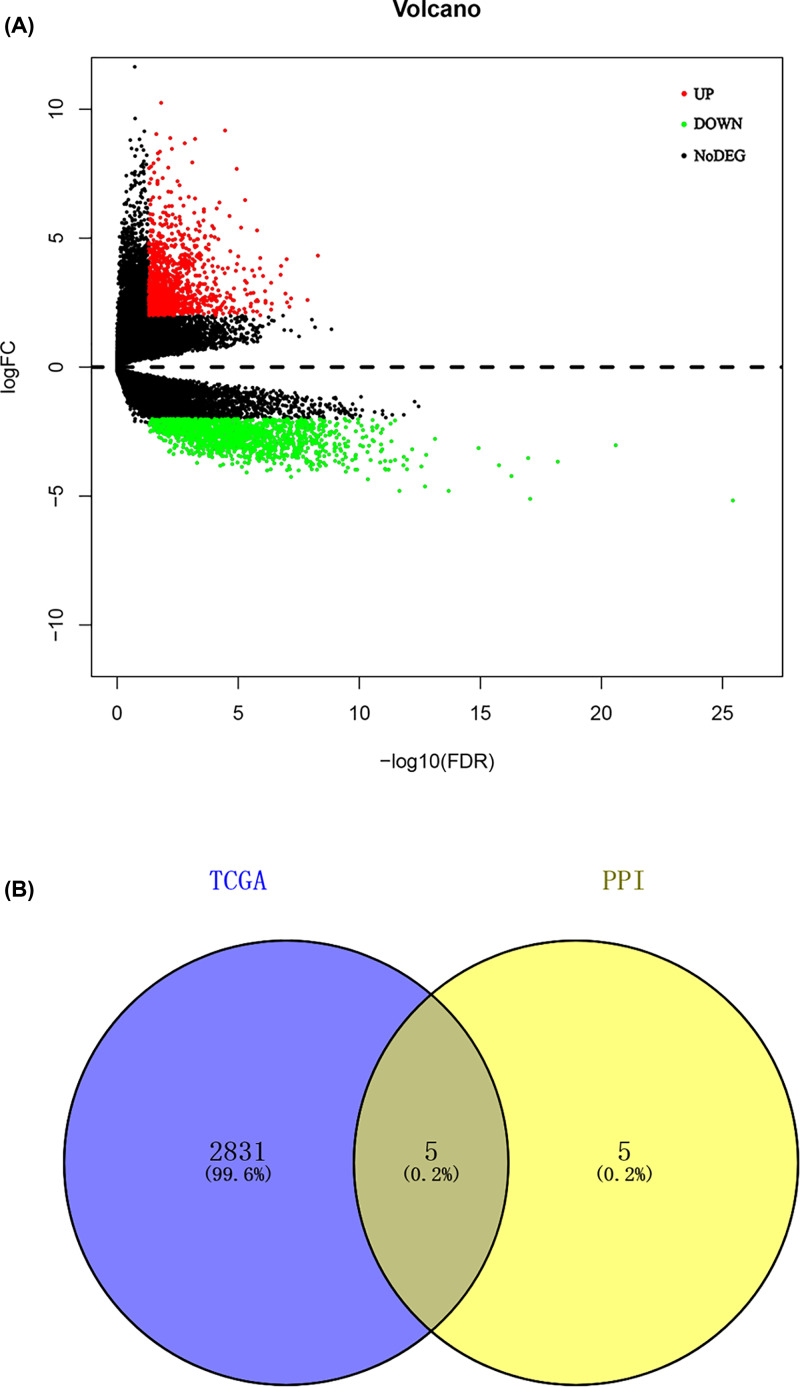
Comprehensive analysis of TCGA and prominent proteins in PPI network **Note:** (**A**) Volcano plot of all DEGs in TCGA. (**B**) Hub genes belonging to both the TCGA and the PPI network.

**Figure 11:**
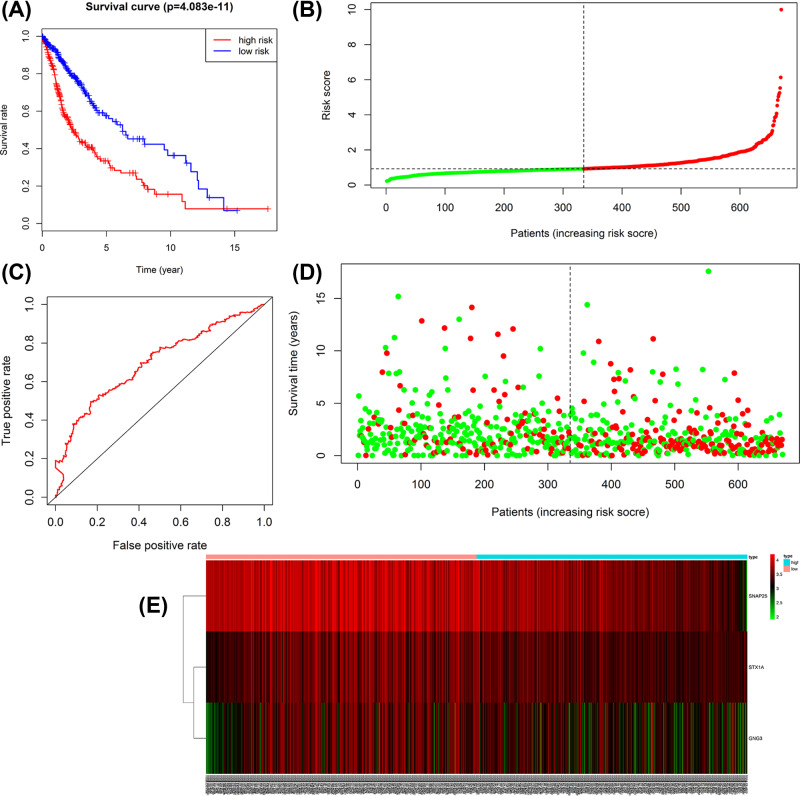
Survival prognosis model of the 6 hub genes **Note:** (**A**) Survival analysis shows that the patients in the high risk grouphas statistically significantworse overall survival than those in the low risk group in TCGA cohort. (**B**) ROC analysis is performed to find out the most optimal cut-off value to divide the GBM patients into the high risk and the low risk groups. (**C** and **D**) The risk scores for all patients in TCGA cohort are plotted in ascending order and marked as low risk (blue) or high risk (red), as divided by the threshold (vertical black line). (**E**) Expression and risk score distribution in TCGA cohort by *z*-score, with red indicating higher expression and light blue indicating lower expression.

**Figure 12:**
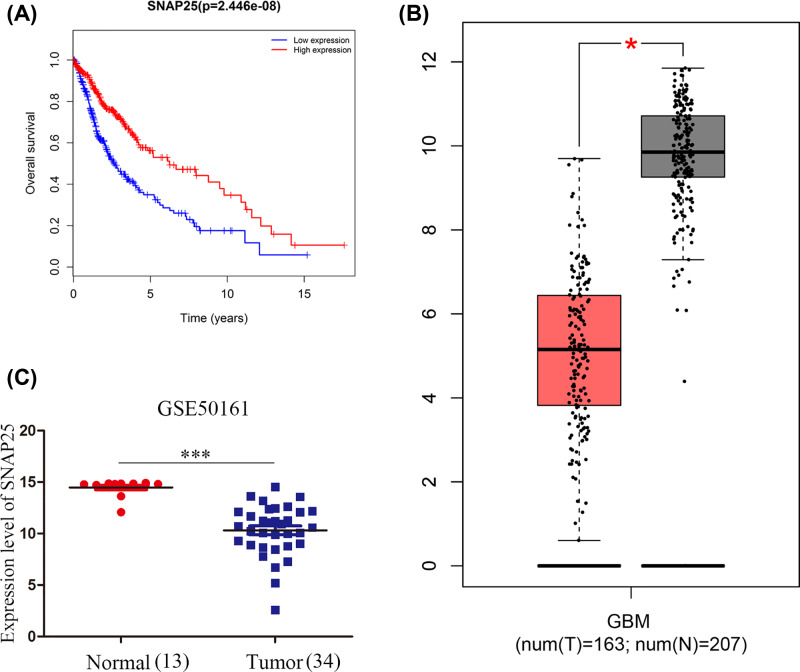
Validation of SNAP25 **Note:** (**A**) Survival analysis of SNAP25 shows that patients with higher SNAP25 expression has better overall survival compared with patients with lower SNAP25 expression. (**B**) Validation of SNAP25 expression in GEPIA shows higher expression in normal tissues compared with tumor tissues. (**C**) Validation of SNAP25 expression in GSE50161 shows higher expression in normal tissues compared with tumor tissues. * and *** represent the difference of SNAP25 expression between normal tissue and glioblastoma tissue.

**Figure 13:**
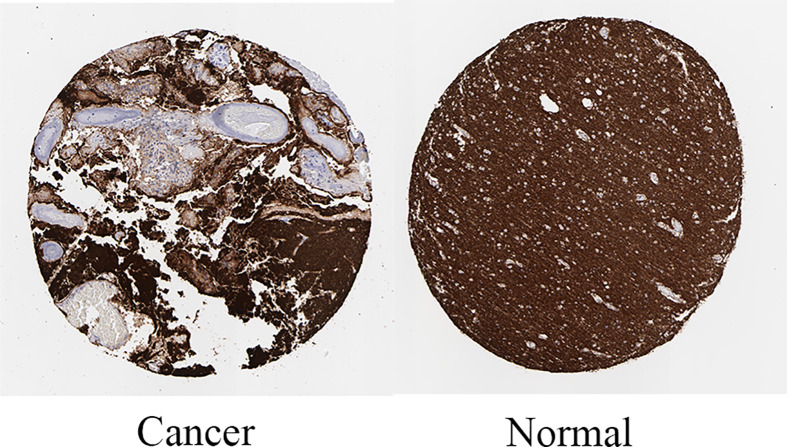
Immunohistochemistry staining of SNAP25 based on the Human Protein Atlas **Note:** Protein levels of SNAP25 in tumor tissues (staining: Low; intensity: Moderate; quantity: <25%; Location: Cytoplasmic/membrane). Protein levels of SNAP25 in normal tissues (staining: Medium; intensity: Moderate; quantity: >75%; Location: Cytoplasmic/membrane.)

**Figure 14:**
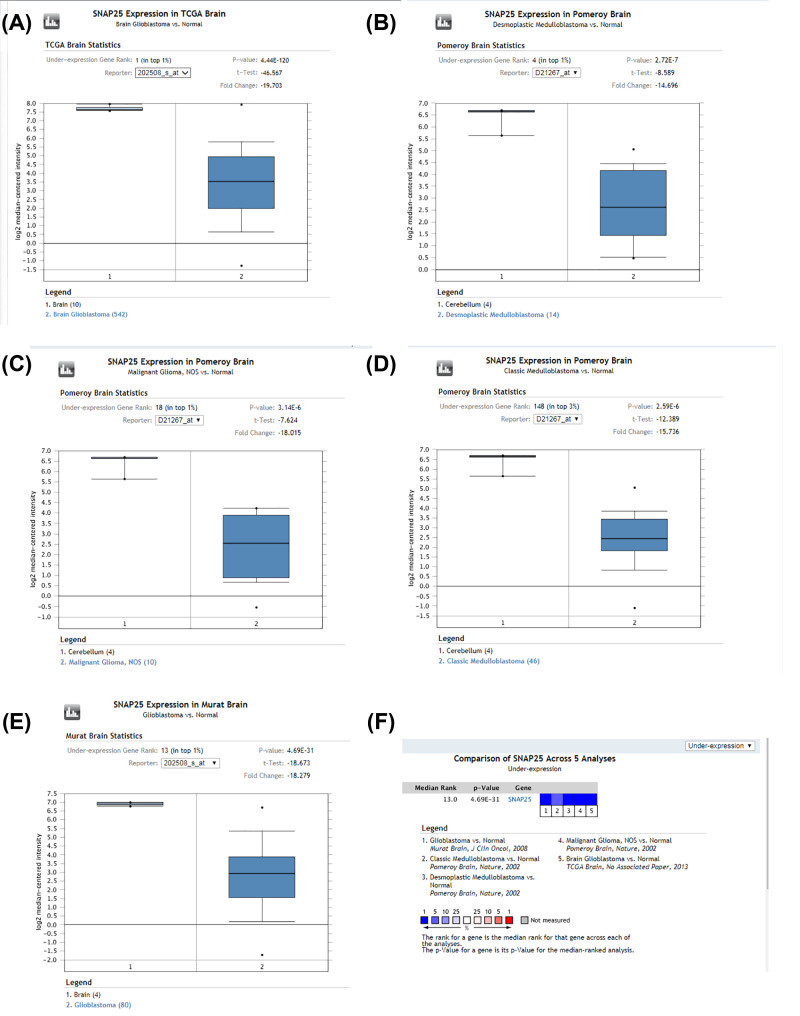
Validation of SNAP25 expression in GBM across multiple datasets by Oncomine **Note:** SNAP25 has the higher expression in normal tissues compared with tumor tissues. (**A-F**) all revealed that expression of SNAP25 was down-regulated in GBM among different analysis datasets,so i put them all together to explain.

**Figure 15:**
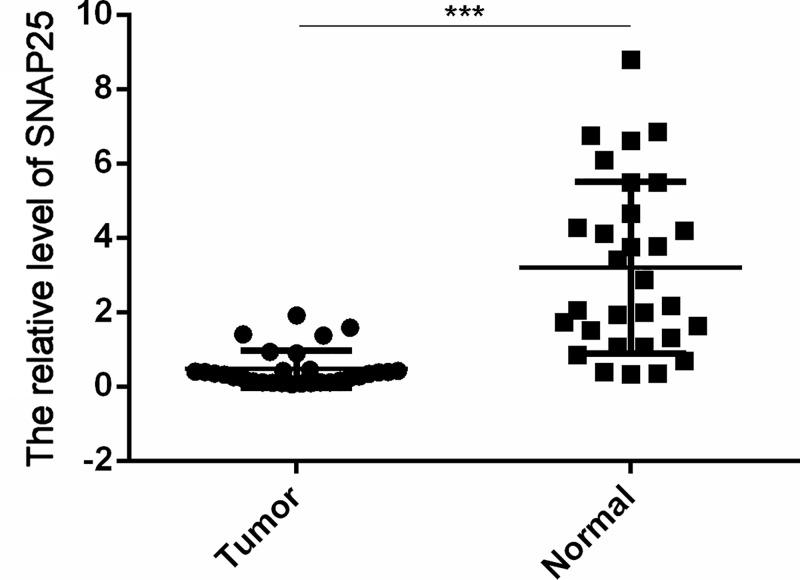
Validation of SNAP25 expression level in clinical tissues by qRT-PCR **Note:** The expression level of SNAP25 is significantly reduced in tumor tissues compared with normal tissues. *** represents the differential expression of SNAP25 in normal tissue and glioblastoma.

The DEGs of TCGA were analyzed using the edegr package with FDR < 0.05 and |log2fold change (FC)| ≥ 2. A total of 2836 DEGs including 1229 up-regulated genes and 1607 down-regulated genes in GBM samples were screened (Figure 10A). The top 10 prominent proteins mentioned in the PPI network and the DEGs in TCGA were comprehensively analyzed. Five hub genes related to GBM, including DNM1, SNAP25, STX1A, GNG3 and SYT1, were screened (Figure 10B) and further coped with univariate and multivariate Cox proportional hazards regression analyses. Three hub genes—SNAP25, STX1A and GNG3—were further screened. The risk score was calculated as follows: Risk score = 0.24*STX1A-0.40*SNAP25+0.15*GNG3. Based on the risk score, GBM patients were divided into the low-risk group and the high-risk group. Kaplan–Meier survival analysis indicated that low-risk patients had significantly better overall survival than high-risk patients in TCGA cohort (Figure 11A). In ROC curve analysis, according to the area of 5-year survival under the receiver operating characteristic curve (AUC), the specificity and sensitivity were both the highest when the risk score was 0.696 (Figure 11B). The distribution of risk score, survival status and the expression of 3 hub genes for each patient were also analyzed (Figure 11C–E). Cox proportional hazards regression analysis showed that SNAP25 was negatively associated with tumor risk. Survival analysis showed that higher SNAP25 expression was linked to better overall survival (Figure 12A). Finally, only the result of SNAP25 was consistent, suggesting that SNAP25 might be the tumor suppressor of GBM. The verification via the GEPIA website showed that SNAP25 was highly expressed in normal tissues (Figure 12B) and lowly expressed in tumor tissues. Immunohistochemistry staining obtained from the HPA database also demonstrated the deregulation of SNAP25 expression in tumor tissues compared with the normal tissues (Figure 13). An Oncomine analysis of cancer versus normal tissue of hub genes in GBM was performed. The results revealed that expression of SNAP25 was down-regulated in GBM among different analysis datasets (Figure 14A–F). Interestingly, the validation of SNAP25 in GSE50161 (including 34 GBM samples and 13 normal samples) demonstrated consistent results (Figure 12C). Finally, the validation of SNAP25 in clinical samples using qRT-PCR showed that the expression level of SNAP25 was significantly reduced in tumor tissues compared with normal tissues, which again proved the conclusion above (Figure 15).

## Discussion

GBM is a common aggressive malignant brain tumor characterized by poor clinical outcomes and short survival time. The mechanism driving its development remains unknown. In the present study, the gene expression profile of GSE22866 in 40 GBM tissue samples and 6 normal brain tissue samples was examined. Then, bioinformatic analysis was used to screen genes that could be used to establish a target treatment.

Using adjusted *P*<0.05 and |logFC| ≥1 as the cut-off, 5505 DEGs (2817 up-regulated and 2688 down-regulated) were identified to be associated with EOC. The upregulated genes were mostly involved in growth factor binding, and the down-regulated genes in gated channel activity. In KEGG analysis, the up-regulated genes were mostly involved in systemic lupus erythematosus, and the down-regulated genes in glutamatergic synapse. These results confirmed the role of DEGs in GBM.

Growth factor binding is found in the progression of various cancers. For example, heparin-binding epidermal growth factor-like growth factor acts as a potent target for breast cancer therapy [23]. Voltage-gated Na+ channel activity also interacts with the transcriptional activity and invasion of colon cancer [24]. Rosenberger et al. found that systemic lupus erythematosus was associated with lung cancer [25]. But, no studies regarding these two molecular processes in GBM have been conducted.

Jung et al. found that VAMP2-NRG1 fusion gene was an oncogenic driver of non-small-cell lung adenocarcinoma [26]. Wang et al. found that microRNA-493-5p promoted the apoptosis and suppressed the proliferation and invasion of liver cancer cells by targeting VAMP2 [27]. In the present study, PPI analysis also identified VAMP2 as the most active protein in GBM.

Comprehensive analysis of PPI network and TCGA, together with survival prognosis analysis, identified that SNAP25 was the real hub gene, and highly correlated with GBM and positively correlated with GBM outcomes. The verification of SNAP25 in GEPIA website showed that SNAP25 was highly expressed in normal tissues compared with tumor tissues. Immunohistochemistry staining demonstrated that the level of SNAP25 was lower in tumor tissues than in normal tissues. An Oncomine analysis of cancer versus normal tissue revealed that expression of SNAP25 was down-regulated in GBM among different analysis datasets. The validation of SNAP25 in GSE50161 and clinical tissues also showed the consistent result.

SNAP25 (synaptosomal-associated protein of 25 kDa) is a membrane-binding protein in neurons [28]. Olbrich et al. found that cleavage of SNAP25 ameliorated cancer pain in a mouse model of melanoma [29]. YanChao et al. found that NUPR1 regulated the late-stage autolysosome processing through the induction of the SNARE protein SNAP25 [30]. The above results showed that SNAP25 might regulate cancer progression. Therefore, the role of SNAP25 in GBM may be illustrated by future research.

However, our research has certain limitations. Our findings should be examined clinically. In the future we will carry out research better designed.

## Conclusion

The present study showed hub functional terms involved in GBM and speculated that SNAP25 might be a tumor suppressor that could be used as a potential biomarker for GBM treatment.

## Data Availability

All data generated or analyzed during the present study are included in this published article.

## Abbreviations

DEG: differentially expressed gene
GEO: Gene Expression Omnibus
GSEA: gene set enrichment analysis
MCODE: Molecular Complex Detection
PPI: protein–protein interaction
STRING: Search Tool for the Retrieval of Interacting Genes Database
